# When Anthropogenic River Disturbance Decreases Hybridisation between Non-Native and Endemic Cyprinids and Drives an Ecomorphological Displacement towards Juvenile State in Both Species

**DOI:** 10.1371/journal.pone.0142592

**Published:** 2015-11-11

**Authors:** Emmanuel Corse, Nicolas Pech, Melthide Sinama, Caroline Costedoat, Rémi Chappaz, André Gilles

**Affiliations:** IMBE, Aix Marseille Université, CNRS, IRD, Avignon Université, Centre Saint-Charles, Case 36, 3 place Victor Hugo, 13331 Marseille Cedex 3, France; Consiglio Nazionale delle Ricerche (CNR), ITALY

## Abstract

Understanding the impact of non-native species on native species is a major challenge in molecular ecology, particularly for genetically compatible fish species. Invasions are generally difficult to study because their effects may be confused with those of environmental or human disturbances. Colonized ecosystems are differently impacted by human activities, resulting in diverse responses and interactions between native and non-native species. We studied the dynamics between two Cyprinids species (invasive *Chondrostoma nasus* and endemic *Parachondrostoma toxostoma*) and their hybrids in 16 populations (from allopatric to sympatric situations and from little to highly fragmented areas) corresponding to 2,256 specimens. Each specimen was assigned to a particular species or to a hybrid pool using molecular identification (*cytochrome b* and 41 microsatellites). We carried out an ecomorphological analysis based on size, age, body shape, and diet (gut vacuity and molecular fecal contents). Our results contradicted our initial assumptions on the pattern of invasion and the rate of introgression. There was no sign of underperformance for the endemic species in areas where hybridisation occurred. In the unfragmented zone, the introduced species was found mostly downstream, with body shapes similar to those in allopatric populations while both species were found to be more insectivorous than the reference populations. However, high level of hybridisation was detected, suggesting interactions between the two species during spawning and/or the existence of hybrid swarm. In the disturbed zone, introgression was less frequent and slender body shape was associated with diatomivorous behaviour, smaller size (juvenile characteristics) and greater gut vacuity. Results suggested that habitat degradation induced similar ecomorphological trait changes in the two species and their hybrids (i.e. a transition towards a pedomorphic state) where the invasive species is more affected than the native species. Therefore, this study reveals a diversity of relationships between two genetically compatible species and emphasizes constraints on the invasion process in disturbed areas.

## Introduction

Understanding how non-native species modify the ecological and genetic features of native biodiversity at local and global scales is a major challenge in conservation biology and of critical importance when dealing with global changes and local exacerbations due to anthropogenic activities [1–2]. Conservation policies stem from various scenarios ranging from species introduction, which is the deliberate or accidental release of species outside of their native ranges, to biological invasions, in which introduced species have already established, spread and present a risk to native species [3–4].

Once non-native and endemic species are in contact many different ecological and/or genomic interactions can occur, the most likely of which is species displacement to a restricted ecological niche due to competition (*e*.*g*. [5]), as illustrated by the competitive exclusion principle [6]. Furthermore, if the interactions concern genetically compatible species, natural hybridisation can happen. In this case, the presence of invasive alleles into the endemic species may lead to its extinction by introgression [7], a situation that has crucial implications for conservation strategies. However, hybridisation may also represent a non-negligible source of adaptive variation in animals, giving rise to new traits (*i*.*e*. transgressive segregation) [8] that can widen the ecological potential of the species (reviewed in [9–10]). Environment and human disturbances are other important factors that may also lead to phenotypic changes and affect the interactions between native and non-native species (reviewed in [11]). For example, river disturbing by human activity is traditionally seen as a major factor imposing constraints because of habitat loss (e.g. spawning site), thereby triggering hybridisation intensity [12–14]. Because these two types of constraints (non-native species pressure and human disturbance pressure) may be simultaneously operating when the impact of invasion is investigated, it is only by including a wide range of conditions that their effects can be dissociated [10].

The impact of non-native on native species is generally assessed by tracking phenotypic changes of the native species in sympatric population. However, studies dealing with various phenotypic traits on specimens obtained from different environmental conditions are seldom in freshwater environment. Moreover, most of the studies on fishes have focused on lake ecosystems (e.g. [15–24]) rather than on stream ecosystems [25–29]. A rare and well-illustrated model of riverine fishes is the non-native *Chondrostoma nasus* (*C*. *nasus*) / endemic *Parachondrostoma toxostoma* (*P*. *toxostoma*), which form several hybrid zones in the Rhône basin in southern France. *C*. *nasus* was found to have a deeper body with a straight mouth, to facilitate epilithon grazing, whereas the endemic species, *P*. *toxostoma*, has a more slender body, with an arched mouth and a more generalist diatom- and invertebrate-based feeding behaviour [25, 30–31]. In the sympatric zone of the Durance River (a left bank tributary of the Rhône River (southern France), both species as well as their hybrids showed common morphological changes (as compared with the reference populations) with a tendency towards a slender body shape and a deformation of the mouth towards a more arched shape [25, 32]. However, the underlying causes of these phenotypic changes remain unclear because the Durance basin presents a particular set of conditions (high fragmentation by hydro-electric dams and weirs, habitat loss, clogging substratum) and is characterized by a mosaic hybrid zone between the native and non-native cyprinid species [25]. Genetic analysis of the same species model was conducted in the largely non-disturbed Ardèche basin, where the hybrid zone corresponds to a tension zone model [33] with most hybrids in the ecotone area [34]. These striking differences among disturbed vs non disturbed systems suggested that hybridisation pattern is strongly shaped by the environment. However, because of the lack of ecomorphological traits comparison between the two systems it has not been possible to establish whether the phenotypic changes observed for both species in the anthropogenic disturbed environment (i.e. Durance basin) resulted from non-native species pressure or from environmental pressure.

The global aim of our study is to assess the impact of the invader *C*. *nasus* on the endemic *P*. *toxostoma* in different areas. We have decided to focus our study on three hypotheses: i) the non native species *C*. *nasus* (considered as invasive) outperforms the endemic species *P*. *toxostoma*. The native species then undergoes trophic niche and morphological traits displacements, inducing a decrease of its “well-being” irrespective of the environment (disturbed or not disturbed) (these changes are not experienced by the non-native species). ii) the proportion of hybrids is higher in the Durance zone (disturbed) than in the Ardèche zone (non-disturbed), due to habitat loss and the overlap of ecological niches. iii) the invader species, which has inhabited the Rhône basin for the past century, represents a risk of extinction for the endemic species by introgression.

This research includes 2,256 specimens from 16 populations (among which the reference populations—where only one species is present—and Rhône basin populations where both species are present). We first conducted a genotyping analysis of all specimens using two sets of markers: the mitochondrial *cytochrome b* gene and 41 nuclear microsatellites. This was coupled with a morphological analysis of the body shape, an investigation of both the coefficient of condition (as a “well-being” specimen index) and the age-size structure of populations, and a non-invasive diet analysis using PCR feces barcoding methods.

## Materials and Methods

### Ethic statement

This study did not have specific approval of a ethical committee on fishes because there was no such committee in 2007–2010 within the Aix-Marseille University. Thus this study was conducted according to relevant national and international guidelines regarding the care and welfare of fishes, following the recommendations of fish ethics of ONEMA (Office National de l'Eau et des Milieux Aquatiques, www.onema.fr/"http://www.onema www.developpement-durable.gouv.fr/".fr/) and DDT (Directions Départementales des Territoires, www.developpement-durable.gouv.fr/"http://www.developpement-durable.gouv.fr/) which are the components of the French state administration. Specimens were caught by electrofishing according to French legislation. After electrofishing, fishes were placed in different storage boxes (arranged in the river). Then specimens were introduced, one after the other, in a tank with aerator and drugs (clove oil corresponding to 10% eugenol, 1 ml for 10 liters) to minimize suffering. After 1–2 minutes in the tank (depending on the size of the fish), the fish was measured, photographed, and scales (for scalimetry), around 10% to 25% (depending of the specimen size) of the caudal fin (for genetic analysis) were sampled. After manipulations, fish was replaced in a new storage boxes arranged in the river till it has awoken. No visible sign of injury or illness due to the manipulation was detected. In our study, all fishes (*P*. *toxostoma* and *C*. *nasus*) were returned to the wild. We stayed on the fishing site during the three hours following the fishes released to detect eventual unhealthy fishes, which are often blocked by the current in the water surface due to rocks, wood-damming or banks obstacles. Involuntary fish mortality represented less than 1.5% of the total sampling (similar proportion of both species) varying in function of campaign (0%-4%) where the highest mortality rate occurred in heat wave period. Recurrent presence of chondrostoms in a same site during the different campaigns during which some already sampled specimens were detected (presence of scare in the caudal fin) suggested that specimens stay alive and healthy after long term period following our manipulations. No other protected or endangered species were involved in the sampling. The field permits specifically included the *C*. *nasus* and *P*. *toxostoma* species (listed on the IUCN Red List [35–36]), cover all fish sampling and experimental manipulations, including the used of clove oil and the size of the fin clip that were reviewed and approved by the ONEMA and the DDT from Alpes-de-Haute-Provence, Hautes-Alpes and Vaucluse (authorizations number 2007–573 and 2008–636), Ardèche (authorization number 2008-91-12 and 2009-112-5), Puy-de-Dôme (authorization number 2007-07/03 and 2008-01-04), Aude (authorization number ONEMA11-2008), and Marne (Authorization number ONEMA51-2007 and ONEMA51-2008).

### Sampling protocol

**Fig 1:**
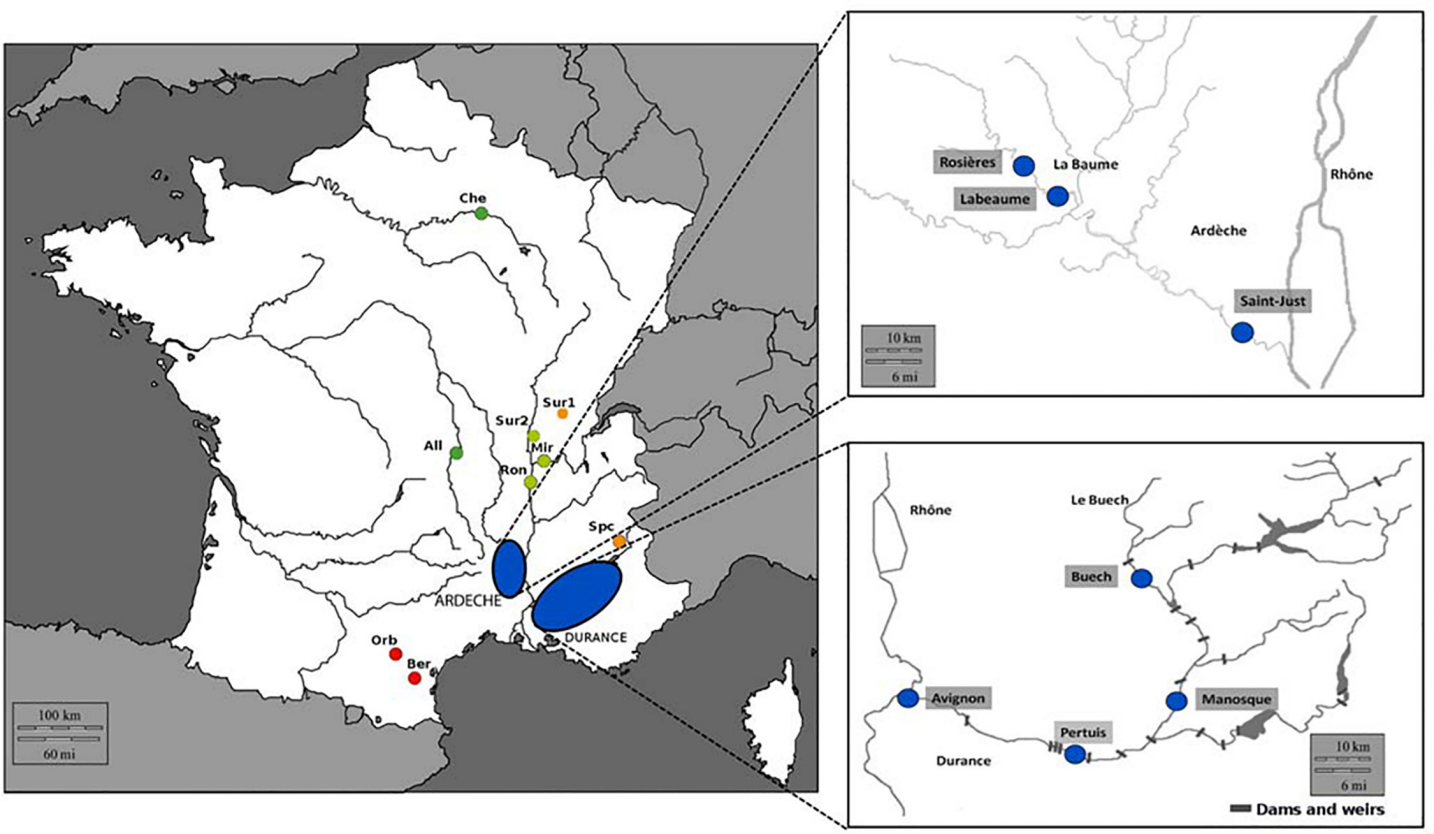
Sampling map. Four allopatric stations (red for *P*. *toxostoma* and dark green for *C*. *nasus*) and 12 sympatric stations (the Rhône basin) were sampled. Furthermore, sympatric populations classified as allotopic stations (five; orange for *P*. *toxostoma* and light green for *C*. *nasus*) and syntopic station (blue stations; three for the Ardèche basin and four for the Durance basin). Allopatric and alltopic populations constituted the reference populations. This picture is a modified version of a copyright free picture of Daniel Dalet, available on www.histgeo.ac-aix-marseille.fr.

Sampling was focused on the *Chondrostoma/Parachondrostoma* complex in allopatric areas (only one species present, with no sign of hybridisation) and sympatric area. Sympatric area is constituted by syntopic (both species, with or without hybridisation) and allotopic (one species, without hybrids, and no physical isolation from the syntopic zones) areas. Allopatric and allotopic populations served as “reference populations” for the syntopic stations. Four populations from allopatric zones corresponding to four different drainage basins were sampled in 2007 and 2008 during spring and summer seasons. During the same period, twelve populations of the two species were sampled from the sympatric zone of the Rhône basin (see Fig 1 and Table 1). This sympatric zone is characterized by different levels of human disturbance and the presence of one to three morphological types of fish (*P*. *toxostoma*, *C*. *nasus* and hybrids). Seven stations presented all three morphologies and constituted two syntopic areas. The first of these areas is located in the Ardèche basin, which had three stations with low levels of fragmentation. The second area was located in the Durance basin, which had four highly fragmented stations (high fragmentation by hydro-electric dams and weirs, habitat loss, clogging substratum) with regulated flows. At these stations, a third sampling was carried out in autumn in order to obtain an accurate description of temporal variations. Five stations presented “pure” specimens on the basis of morphological identification and defined potential allotopic population to be confirmed by molecular analysis. A piece of caudal fin and feces were sampled and fixed in alcohol, and placed at -20°C for long-term storage. Each specimen was measured and weighed before scales were collected for age determination.

**Table 1:** Number of specimens considered, by analysis. All specimens were genotyped, and only subgroups of specimens were used in the various analyses, because size, weight measurements, images of the body or feces was unavailable.

Zone		Station	Latitude	Longitude	Genotyping	K	Morpho	Diet (DNA barcoding)
Allopatry	*Pt*	Ber	43° 1' 58''N	2° 49' 54''E	*44*	43	*39*	**0**
		Orb	43° 6' 50''N	2° 37' 24''E	*133*	**115**	*114*	**116 (15)**
	*Cn*	Che	48° 47' 38''N	4° 51' 31''E	*93*	**93**	*73*	**96 (32)**
		All	45° 37' 91''N	3° 12' 48''E	*96*	**84**	*84*	**84 (43)**
Allotopy	*Pt*	Spc	44° 32' 11''N	6° 25' 31''E	*36*	**0**	*0*	**0**
		Sur1	46° 15' 52''N	5° 25' 46''E	*79*	**78**	*69*	**78 (21)**
	*Cn*	Sur2	46° 2' 55''N	5° 19' 27''E	*37*	**0**	*0*	**0**
		Ron	45° 22' 18''N	4° 45' 14''E	*40*	**0**	*0*	**0**
		Mir	45° 49' 17''N	4° 57' 19''E	*33*	**0**	*0*	**0**
Syntopy	Ardèche	Ros	44° 28' 30''N	4° 15' 51''E	*172*	**169**	*157*	**166 (11)**
		Bau	44° 26' 56''N	4° 18' 31''E	*142*	**115**	*111*	**113 (25)**
		Jus	44° 17' 41''N	4° 36' 51''E	*134*	**103**	*129*	**103 (22)**
	Durance	Bue	44° 20' 40''N	5° 46' 13''E	**322**	**307**	**295**	**295 (65)**
		Man	43° 55' 32''N	5° 53' 58''E	**395**	**379**	**336**	**376 (56)**
		Per	43° 40' 50''N	5° 29' 36''E	**142**	**126**	**122**	**126 (22)**
		Avi	43° 54' 48''N	4° 49' 14''E	**356**	**354**	**287**	**346 (63)**
Total					**2,256**	**1,966**	**1,816**	**1,896 (375)**

K = coefficient of condition, Morpho = morphological analysis of the body, Diet = vacuity analysis and in parenthesis the DNA barcoding sub-sample. *Pt* = *P*. *toxostoma*, *Cn* = *C*. *nasus*, Sur = Suran, Ber = Berre, Orb = Orbieu, Che = Chée, All = Allier, Mir = Miribel, Ron = Roussillon, Ros = Rosières, Bau = Labeaume, Jus = Saint-Just, Bue = Buech, Man = Manosque, Per = Pertuis, Avi = Avignon, Spc = Serre-Ponçon. Data previously published are in italics [29,34], new data are in bold.

### Genetic identification

Unless otherwise specified, all statistical analyses were carried out with R version 3.0.2 [37]. Total genomic DNA was extracted from the caudal fin sample, and a 500-bp fragment of the mitochondrial cytochrome *b* gene (which can be used to determine the maternal inheritance of individuals) was sequenced by standard polymerase reaction (PCR) procedures, as previously described [38]. Seqscape 2.5 software (Applied Biosystem®) was used to align and correct sequences. We amplified 41 microsatellite loci with five multiplex PCR kits, as described in [34] and [39]. The Introgress R package [40–41] was used to estimate the hybrid index (*h*). A second hybrid index was estimated using the Bayesian model-based clustering algorithm implemented in the software Structure 2.3 [42]. As both indices are highly correlated (S1 Fig), only the *h*-introgress index was considered in our study.

Polymorphism shared by the two species was taken into account by performing the Introgress analysis, first on allopatric populations and then on allopatric + allotopic populations, as described in [34]. This made it possible to define ranges of *h* corresponding to pure specimens: *C*. *nasus* specimens had *h* values in the range [0–0.086], whereas *P*. *toxostoma* had *h* values in the range [0.934, 1]. We therefore assigned individuals to three classes, as follows: *Cn* if *h* was in the range [0, 0.086], *Pt* if *h* was in the range [0.934, 1] and Hybrid if *h* was in the range (0.086, 0.934). A nuclear *Cn* (resp. *Pt*) individual presenting *Pt* (resp. *Cn*) mtDNA is considered as hybrid.

### Ecological traits

#### Age, size and coefficient of condition

We compared the mean sizes of the groups (*C*. *nasus*, *P*. *toxostoma*, *hybrids*) between three environment conditions (Durance basin, Ardèche basin and “reference populations,” variable env), performing ANCOVA (type III tests). Age, determined by scalimetry, was used as variable of adjustment for mean size comparisons.

Coefficient of condition K (= weight (in mg)/size (in cm)^3^) was considered as an estimate of the "well-being" of the fish [43]. We studied the effect of three factors on *K*: size, species status (*Pt* or *Cn*), designated *sp*, and environment condition (env).

We first consider a dataset (n = 1,809) corresponding to non-hybrid specimens after the exclusion of 12 specimens because they present extreme *K* values (K<5 or K>20), most likely due to errors in the determination of weight. This dataset included individuals from all sets of conditions (the Durance basin, the Ardèche basin and the “reference populations,” defining the env variable). We considered the model K = env x bs(size, m) x sp + ε where bs (size, m) is the polynomial basis of degree m*<6* for size, and the value of *m* is selected on the basis of the Akaike information criterion (AIC). In the selected model, a type III sum of squares was considered in order to test the effects. The interpretation of simple effects was simplified by centering the size variable (centered) on the overall median (13.25 cm). For example, species comparisons were carried out for a fixed size of 13.25 cm. We then considered hybrid specimens (n = 145). As hybrid specimens are genetically defined by h, they may be theoretically viewed as a mixture of a *P*. *toxostoma* specimen (proportion h) and a *C*. *nasus* specimen (proportion 1-h) with similar characteristics (size, environment). We then tested the hypothesis that the hybrid K value is a linear mixture of the parental K values by comparing the observed K for the hybrids with that predicted theoretically assuming a linear mixture of the two pure species. For each specimen, the difference D between these two values was studied considering the following model:

D=env×size×h+ε

The env variable here can take only two values: "Ardèche" and "Durance," as this analysis is restricted to hybrid specimens.

#### Diet analysis

*Feeding activity*- The effects of h, season, size and environment on gut vacuity (Guvac = presence/absence of feces) were investigated via the following logistic model:

logit(P(Guvac=1))=h×(season+size+env)

*Feeding behaviour-* IUCN Red List states *P*. *toxostoma* as vulnerable A2ce ver 3.1 [35] in their origin area and the *C*. *nasus* as least concern ver 3,1 locally threatened [36]. Dietary analyses therefore had to be carried out by non-invasive methods. We therefore used the PCR-based method for feces developed in a previous study [31] to obtain qualitative (presence/absence) information about the prey ingested by the fish (S1 Table). We then construct a feeding index defined by the first axis of the PCA on prey proportion [44–45]. This index, after discretization, was subjected to a multinomial logistic modelling in which season, environment and species defined the explanatory variables.

#### Morphological analysis

We used the landmark-based morphometric approach as described in [25]: 21 homologous landmarks were digitized with TpsDig software [46], and a generalized procrustes analysis (GPA) was performed on their coordinates. We thus considered 21 x 2 = 42 morphological variables. Each coordinate was then modeled in the same way as the coefficient of condition K. First we analyzed non-hybrid specimens (n = 1,684), and second we compared hybrid specimens (n = 132) to predictions obtained via the first model (S1 Text). Explanatory variables are species status *sp* (*Pt* or *Cn*), environment conditions (env) and size. Size is considered here as a surrogate for the development. Hence morphological modifications related to the size will be interpreted as ontogenetic deformations [47]. All the data produced for this work are available on S2 Table.

## Results

### Molecular analyses

Molecular results (data not shown) confirmed the pre-supposed populations status: first the a priori allotopic populations presented effectively only one species (*C*. *nasus* or *P*. *toxostoma*) and hybridisation was never observed, second a priori syntopic populations (four stations of the Durance basin and three stations of the Ardèche basin) presented systematically both species with hybrids. From this result it was possible to distinguish reference populations (*ie* allopatric and allotopic) from syntopic populations.

Considering nuclear data, the percentage *of P*. *toxostoma* specimens ranged in the Ardèche basin from 33.58% in St Just (downstream part of the river) to 80.47% in Rosières (upstream part of the river) (S2 Fig). By contras*t*, *C*. *nasus* ranged from 59.70% in St Just to 2.37% in Rosières. The upstream-downstream gradients of the two species were thus opposite and non-symmetric. Conversely, in the Durance, the middle section of the river was mainly dominated *by P*. *toxostoma* (95.74% at Pertuis and 87.34% at Manosque), whereas the upstream and downstream parts of the river were dominated *by C*. *nasus* (57.76% at Buech and 80.23% at Avignon).

All syntopic samples included hybrid specimens (Ardèche and Durance basins, S2 Fig) and can be split into two classes. The first class contains a maximum of 6.72% hybrids, observed in the Durance basin and Saint-Just (the most downstream populations of the Ardèche).The second class corresponds to a higher percentage of hybrids, 17.16% at Rosières and 39.72% at Labeaume, the two upstream populations of the Ardèche basin. Hence, the hybridisation rate appear to be homogeneous at low levels in the Durance basin and heterogeneous, including at high levels, in the Ardèche basin. Distribution of h (S2 Fig) is skewed on values higher than 0.5 in the direction of *P*. *toxostoma* (h = 1) indicating that introgression tended to involve the transfer of genes from *C*. *nasus* to *P*. *toxostoma*. There is two exceptions concerning the Avignon and Buech populations, in which *h* appears to be centered around 0.5.

### Size and age

Taking into account all the stations on the Durance and Ardèche basins, the mean size of *C*. *nasus* in the Ardèche was below that in the Durance and reference populations (*P* = 0.005). However, this result was strongly influenced by the downstream stations (i.e. Saint Just and Avignon). Indeed, the spring sampling at the Saint-Just station followed a flooding event, and many small specimens were detected. By contrast, the Avignon sampling campaigns were systematically characterized by the presence of large *C*. *nasus* specimens absent from the rest of the upstream part of the Durance. Such individuals may be related to the Rhône environmental conditions, as the Avignon station is near the Rhône River. When excluding these populations, both species were, in comparison to reference populations, longer in the Ardèche (Δ_*C*.*nasus*_Size = +3.02 cm, *P* = 0.01 and Δ_*P*.*toxostoma*_Size = +1.60 cm P = 1.74e-11) and smaller in the Durance (Δ_*C*.*nasus*_Size = -3.86 cm, *P* = 2.21e-15 and Δ_*P*.*toxostoma*_Size = -2.33 cm *P* = 2e-16) (S3 Table). Similarly, hybrids in the Durance were smaller than those in the Ardèche (Δ_*hybrids*_Size = -2.35 cm, *P* = 2.11e-4). This model is adjusted for age, highlighting the smaller size of specimens in the upstream and middle sections of the Durance, regardless of the group considered (*C*. *nasus*, *P*. *toxostoma* or hybrids). This result indicates a general trend towards a lower growth rate in this zone.

### K coefficient

In allopatry, no significant difference in mean *K* values was found between the two species (S4 Table). However, a significant effect of the Durance was detected (Δ*K* = -1.51; *P* = 1.62 10^−13^), corresponding to a lower *K* in this zone, particularly for *C*. *nasus*. We also identified a negative relationship betwe*e*n K and the size *of P*. *toxostoma* (S3 Fig) in the Durance and Ardèche environments. This relationship was positive in allopatric condition*s* (P<0.001). Hybrids whose h-value is around 0.5 presented higher K-values than predicted by the model, particularly for the Ardèche basin (S3 Fig).

### Diet analyses

*Feeding activity analys*i*s- C*. *nasus* has a significantly (P = 0.02) higher probability of having feces th*an P*. *toxostoma*. Furthermore, probability to detect feces was significantly (*P* = 9 x 10^−5^) higher in reference populations than in syntopic conditions, indicating that specimens display lower levels of feeding activity in syntopic conditions (S4 Fig and S5 Table). This is particularly true for *C*. *nasus*, which presented greater differences with respect to reference populations. An interaction between period and *h* was also detected (*P* = 1.7 x 10^−4^), with higher levels of feeding activity in the spring than in the summer and autumn, given that specimens tended to be *P*. *toxostoma* (S4 Fig and S5 Table).

**Fig 2:**
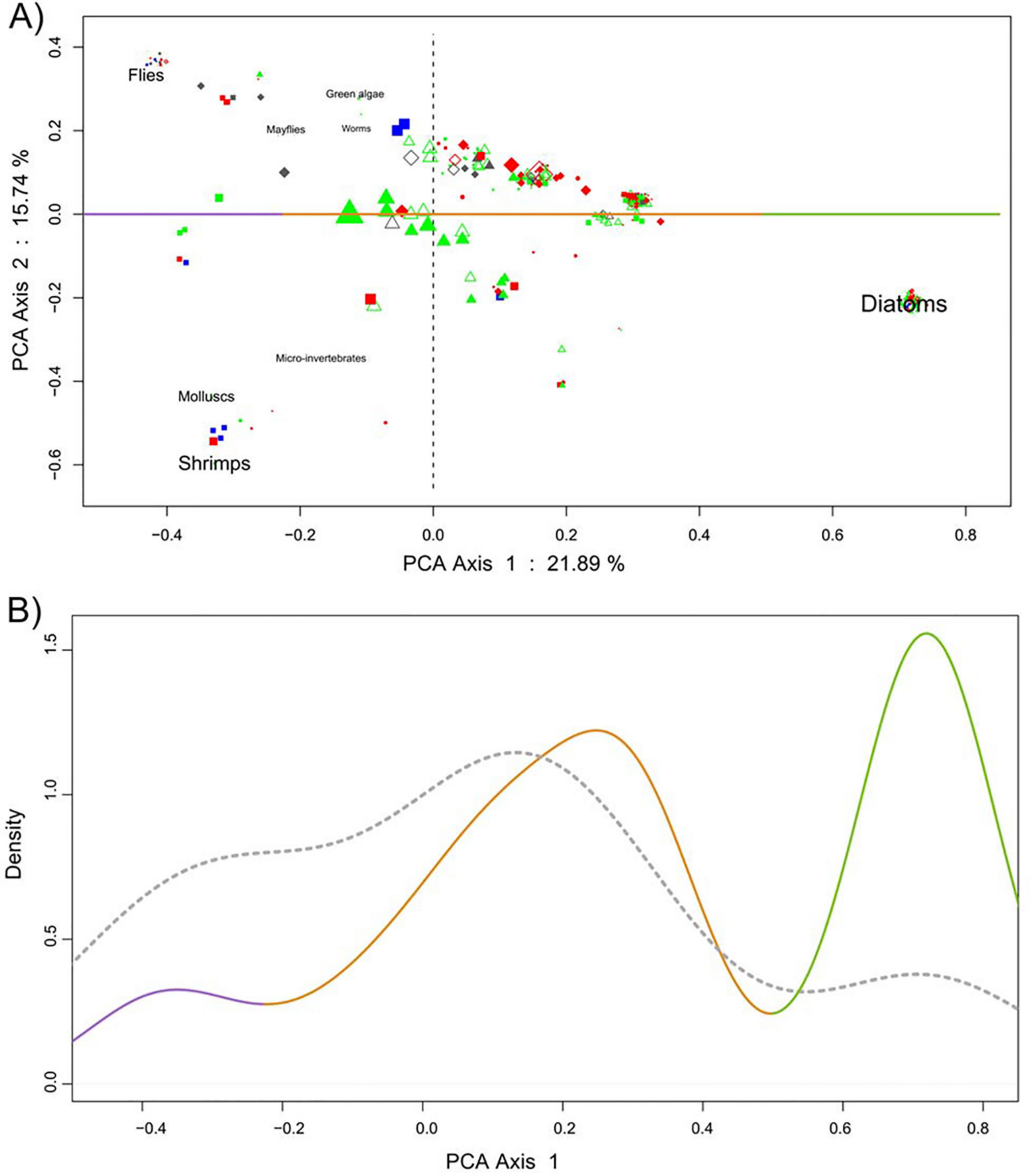
Feeding behaviour. Molecular detection in the feces of the eight prey items analyzed by a principal component analysis of proportions. **A)** Principal plane of the PCA. The reference populations are represented by closed triangles for the Chée River (Cn. allopatry), open triangles for the Allier (Cn allopatry), closed diamonds for the Suran (Pt allopatry), and open diamonds for the Orbieu (Pt allopatry). The syntopic zones are indicated by a closed square for the Ardèche basin and a closed circle for the Durance basin. *C*. *nasus* is shown in green, *P*. *toxostoma* in red, hybrids in blue and *Barbus barbus* (insectivorous control) in grey. The size of the symbols for each specimen is proportional to the number of prey items detected. **B)** Non-parametric density estimation (Gaussian kernel) of the first axis co-ordinates. Each color corresponds to a category as follows: in purple, invertebrate eaters (i.e. only invertebrates detected in feces); in orange, omnivores (both invertebrates and diatoms detected); and, in deep green, diatom eaters (only diatoms were detected). The distribution density corresponding to *Barbus barbus* specimens is indicated by grey dashes.

*Feeding behaviour-* We analyzed the feeding behaviour of chondrostoms based on the identification of eight prey items corresponding to eukaryote preys. In PCA (see Fig 2A), the first axis accounted for 21.89% of the total inertia and is strongly correlated with the proportion of diatoms (R^2^ = 0.97*; P<10* ^*−6*^ *)*. It clearly opposed the “diatoms” prey item to the “invertebrate” prey item formed by Diptera, Mollusca and Malacostraca. The three modes of specimen distribution along this axis (Fig 2B) allowed the identification of three classes of prey detection patterns: invertebrate eaters (8.62% of the total distribution), omnivorous behaviour (53.64% of the total distribution) and diatom eaters (37.74% of the total distribution).

**Fig 3:**
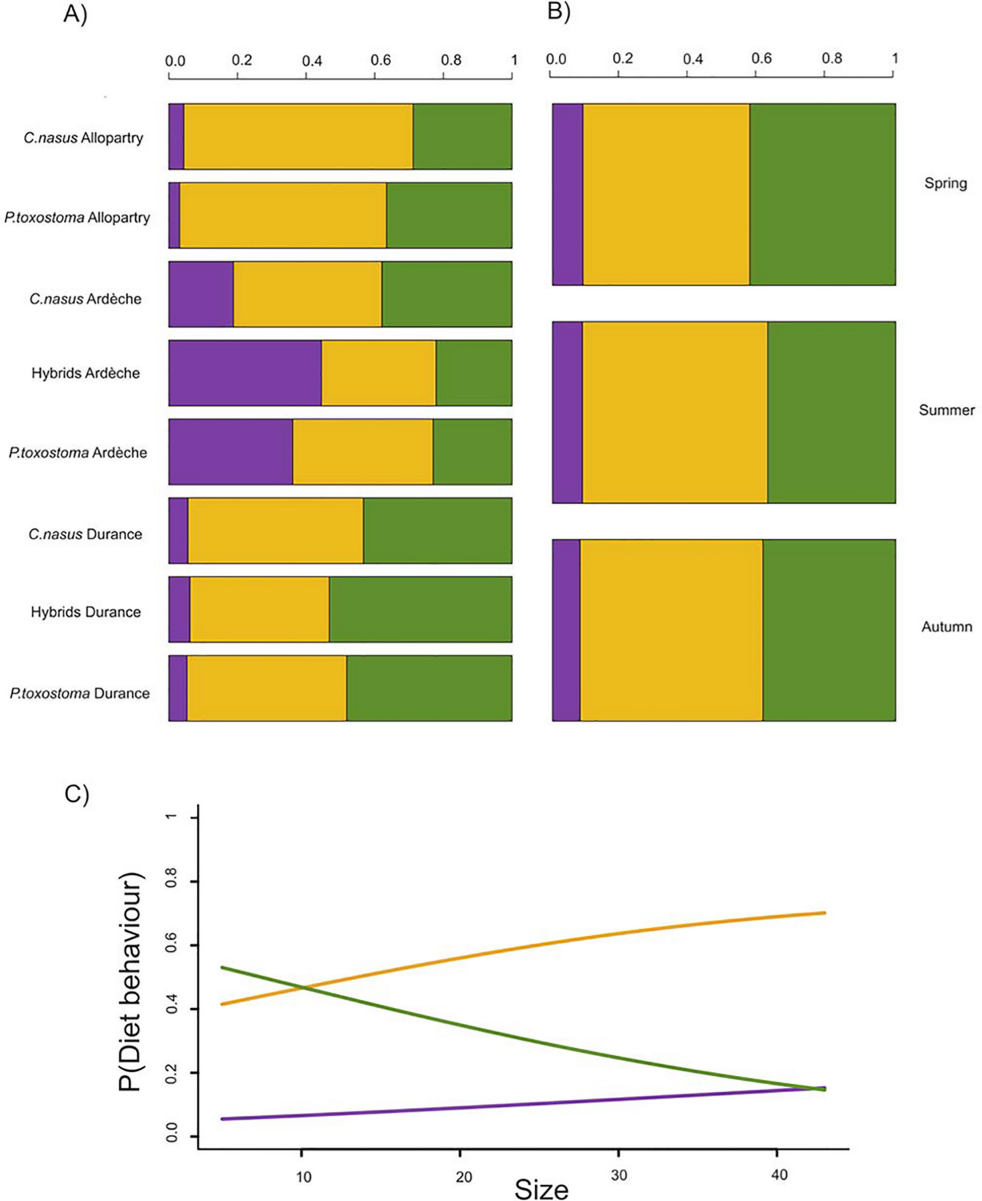
Influence of group, environment, season and size on feeding behaviour. Distribution of the three classes of feeding behaviour (invertebrate eaters in purple, omnivores in orange and diatom eaters in deep green). Stacked bar chart showing the proportions of the various feeding behaviour classes by species and zone type in **A**), by season for each syntopic zone in **B**). Probability of GutVac (*y*-axis) as predicted by the multinomial model as a function of size in **C)** in which the *X*-axis corresponds to body size in cm, and we can read for example the longer specimens are more omnivor than herbivor.

The multinomial logistic model explaining the three classes (invertebrate eaters, omnivorous and diatom eaters) highlighted a strong effect of environment (χ^2^ = 36.97, df = 4, *P*<10^−6^), with the reference populations being more omnivorous (regardless of species), the Ardèche containing more invertebrate eaters and the Durance, more diatom eaters (Fig 3A). There appeared to be a seasonal effect, with more diatom consumption in the spring than in the other seasons (χ^2^ = 11.30, df = 4, *P* = 0.02) (Fig 3B). Size also had a strong effect on feeding behaviour (χ^2^ = 13.28, df = 1, *P*<10^−6^ **)**, as diatom consumption was associated with smaller size (Fig 3C). Finally, we did not detect differences in feeding behaviour between the two species and their hybrids (*P* = 0.73). Furthermore, the hybrid specimens displayed a tendency to behave differently between the two rivers, with more diatom eaters in the Durance and invertebrate eaters in the Ardèche.

### Morphological analysis

**Fig 4:**
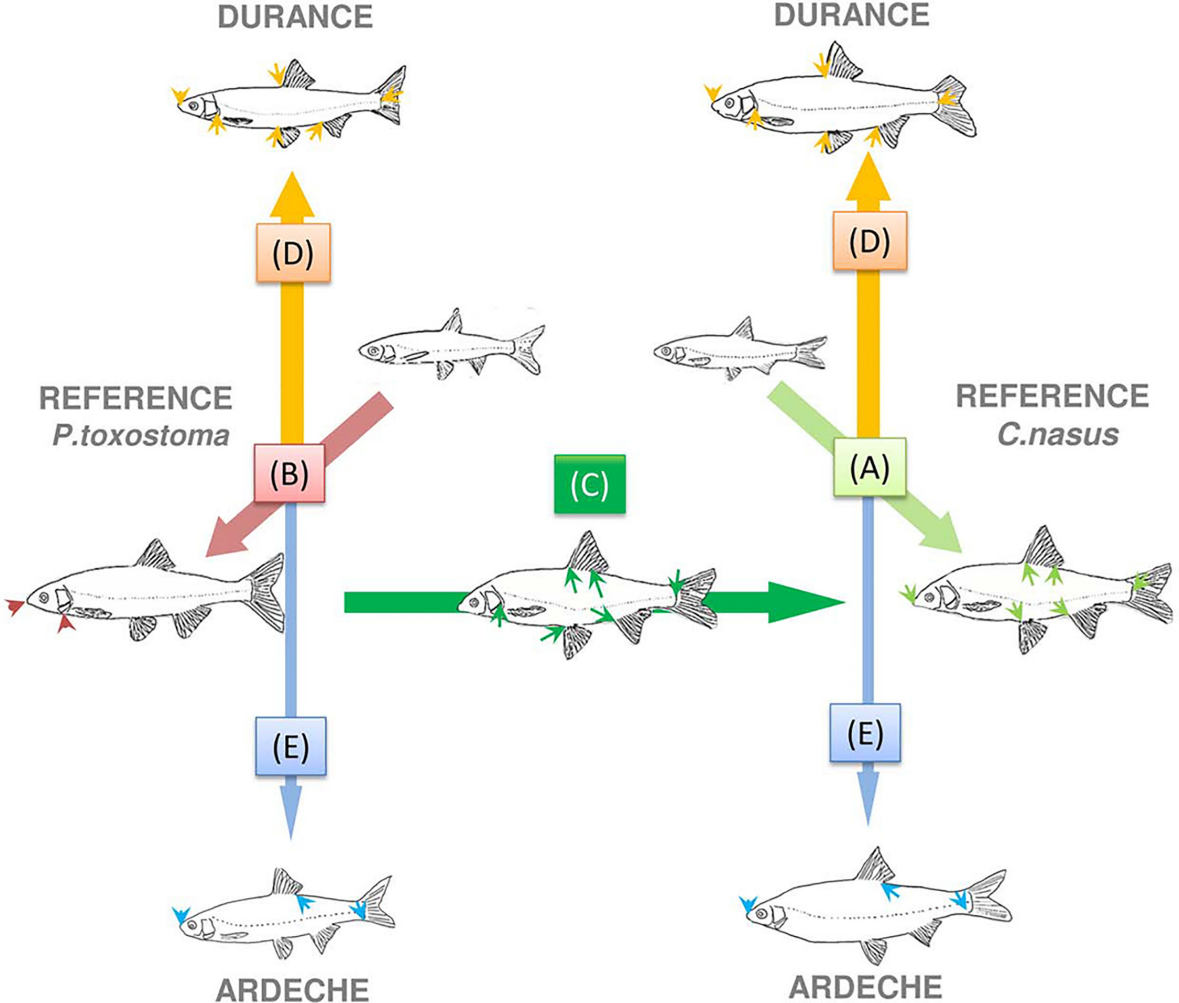
Summary of body deformations taking into account species, size, environment and their interaction. A) Ontogenic deformations f*or C*. *nasus* in reference condition, B) Ontogenic deformations f*or P*. *toxostoma* in reference condition, C) Interspecies deformations in reference condition, D) Deformation from reference condition to Durance for both species, E) Deformation from reference condition to Ardèche for both species. See S5 Fig. for more details. The width of an arrow is positively related to the intensity of the deformation.

The intensity of ontogenetic deformations, based on specimens ranging from 10 cm (for both species) to 32.4 cm in *C*. *nasus* and 18.5 cm in *P*. *toxostoma* (Fig 4 and S5 Fig), differed between the species and was generally stronger for eight landmarks (landmarks 2, 4–7, 9 and 10, Fig 4 deformation A) in *C*. *nasus*. The greater range of sizes in *C*. *nasus* (maximum size of up to 32.4 cm) than in *P*. *toxostoma* exacerbated the morphological differences between the two species. *C*. *nasus* moves from a slender to a deeper-bodied morphology (with a ventral positioning of the snout and a dorsal positioning of the caudal peduncle) with increasing body size. By contrast, *P*. *toxostoma* displayed a slender morphology that was accentuated during the ontogeny of this species (due to the upward movement of landmarks 12 and 20, Fig 4 deformation B). The species differences between *P*. *toxostoma* and *C*. *nasus* corresponded essentially (but not exclusively) to the ontogenetic deformation of *C*. *nasus*, suggesting multivariate allometric differences (Fig 4 deformation C).

We detected no common syntopic effects in the Ardèche and Durance basins with respect to the reference populations, but a clear Durance effect was observed (Fig 4 deformation D). This effect essentially involved four landmarks (4, 5, 7, 9, 10 and 19), indicating a general tendency for the fish from the Durance to be more slender, with landmarks 4 and 5 converging on landmarks 9 and 10. The Ardèche effect (Fig 4 deformation E) was in comparison weaker and concerns only few points (2, 6 and 8).

Differences observed between hybrids from the Ardèche and hybrids from the Durance, as illustrated in S6 Fig, were similar to the deformations observed for pure specimens from the Durance with respect to reference populations (S6A Fig), with a more slender shape (see pelvic and dorsal fin landmarks). Thus, the Durance effect acted on all specimens, regardless of their genotypes. Moreover, the shapes of the Ardèche and Durance hybrids matched expectations (S6A, S6B and S6C Fig), with morphological deformation along hybridisation index corresponding to a linear model of transition between the shapes of the two pure specimens.

## Discussion

**Fig 5:**
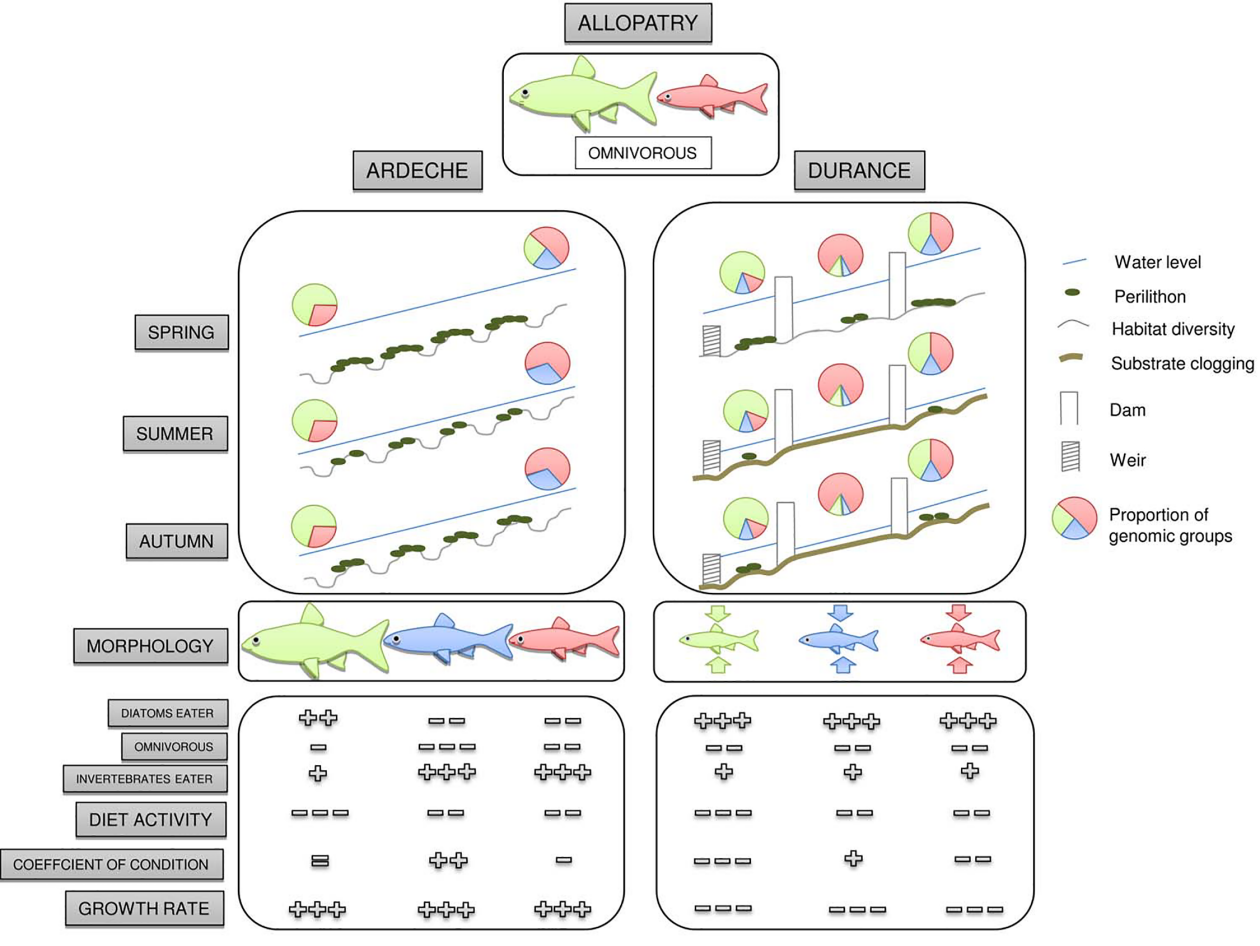
Integrative ecological pattern for the *Chondrostoma* complex in the two syntopic zones. Environmental parameters, such as water level, perillithon availability and clogging, are shown diagrammatically, with the distribution of the three genomic groups over the upstream-downstream gradient of the rivers. Morphological analysis, feeding behaviour, feeding activity, the coefficient of condition and growth rate are shown schematically in the three boxes. In the Ardèche, the responses of the three genomic groups differed. By contrast, in the Durance basin, the three genomic groups displayed a similar tendency.

The aim of this study was to assess the impact of a non-native species on a native one in different environments. We monitored ecomorphological traits changes in two french chondrostom species inhabiting disturbed (Durance River) and non disturbed (Ardèche River) sympatric areas in the Rhône basin (southern France). Our results (summarized in Fig 5) showed a non homogeneous pattern between the two areas, i.e. we observed important traits changes only in the disturbed river for both species. Genomic analyses also showed differences in the hybridisation patterns and intensities between the two zones. Together, these results suggest that the disturbance of the rivers resulted in a strong environmental pressure shaping the phenotypes and the interactions of both species and their hybrids.

### Species coexistence in a non-fragmented environment (Ardèche basin)

As previously reported [34], the population dynamics observed in the Ardèche basin follows a sigmoid tendency (with *P*. *toxostoma* mostly at the upstream Rosières station, *C*. *nasus* mostly at the downstream Saint-Just station and hybrids mostly at the ecotone Labeaume station). However, the presence of hybrids (with *C*. *nasus* mtDNA) in the upstream part of the river seems to indicate a migration of female *C*. *nasus* to the reproductive zone of *P*. *toxostoma* during the spawning period in spring and/or to the existence of an hybrid swarm (see Fig 5). This hypothesis is supported by the presence of few pure *C*. *nasus* specimens in this zone, regardless of the period. Indeed, in non-fragmented rivers, *C*. *nasus* is known to inhabit mostly deep habitats in wide sections of the river or in the downstream part of tributaries and to migrate upstream to spawn [48–50]. By contrast, *P*. *toxostoma* is known to inhabit tributaries or medium-sized rivers and to migrate short distances to spawn [51–52]. The presence of some hybrid specimens in the Saint-Just station (the downstream part) could be explained either by a low selection pressure against hybrids (s) or by a large migration of “pure” specimens (m). These observations force a reappraisal of the delimitation of the hybrid zone as described in [33], where the sigmoid distribution (involving the presence of only one species at each extremity) is defined strictly on the Ardèche basin. In accordance with the sigmoid model, the Ardèche hybrid zone should rather be extended downstream to the Saint-Just station, in the Rhône River, where only “pure” *C*. *nasus* specimens can be found.

Compared to what is observed in allopatric populations, Ardèche’s *P*. *toxostoma* displayed more insectivorous behaviour, a higher vacuity coefficient but a similar body morphology. The *C*. *nasus* specimens presented similar eating behaviours to *P*. *toxostoma*. In other words, the two species and their hybrids all underwent feeding behaviour changes towards a more insectivorous diet compared to the reference populations. This particular feeding behaviour may be linked to the documented high invertebrate density (i.e. high prey availability) in this river [53].

### Diet modulation based on the ontogenetic plasticity in disturbed environment (Durance basin)

The hybridisation dynamics observed in the Durance basin have strikingly lower and more uniform rates of hybridisation along the upstream-downstream gradient (3.35–5.90%) than in the Ardèche basin. Another difference between the two areas is that the specimens of both species from the Durance River were younger and smaller than those of the reference populations. These results are consistent with patterns observed in 2001 and 2002 [25]. The syntopic specimens had a lower *K* value (particularly for *C*. *nasus*), a higher vacuity coefficient, a higher proportion of diatoms in their diet and a more slender body shape than allopatric specimens of similar size. *K* value, gut vacuity coefficient and deeper-bodied deformation (for *C*. *nasus*) are positively related to size, and size is negatively related to diatom consumption. Hence, the Durance specimens clearly presented the characteristics of smaller allopatric specimens. Because the Ardèche populations did not display such a pattern, we assume that this ecomorphological pedomorphosis did not result from syntopic interactions between species. As a consequence, the hard river engineering of the Durance basin seems to be a more likely explanation. Previous studies have already shown how fish communities are disturbed in Durance [54–55], favoring opportunist rheophilic and short length species [54]. These studies assumed that the expected zonation of grayling-barbel [56] has been replaced by a vairone (*Telestes souffia*, Risso 1827)—spirlin (*Alburnoides bipunctatus*, Bloch 1782) zonation. The environment, particularly in terms of warm temperatures, habitat loss, flow exposure and substrate clogging, may have affected both species.

The temperature of the water in the Durance basin differs considerably along the upstream-downstream gradient. Indeed, the number of degree-days >20°C is 50.75+/-20.31 at Buech, 53.00+/-19.88 at Manosque, 134.50+/-26.32 at Pertuis, and 107.75+/-23.22 at Avignon (data from 1995 to 2002; from upstream to downstream [25]). These high temperatures may have a particularly strong impact on chondrostoms, due to their upper optimal temperature limits (25°C for *P*. *toxostoma* and 24°C for *C*. *nasus*) [55,57]. Moreover, during summer, the Mediterranean regime is characterized by a very dry period [58], followed or preceded by stochastic strong flooding events, which kept happening in the Durance River despite flow regulation, though at lower frequencies [59–60]. Such flood events, added to habitat loss, increase exposure to currents and promote body shape changes by positively selecting a more hydrodynamic morph [61–62]. The slender deformation observed for the whole complex (pure specimens and hybrids) in the Durance may therefore enhance hydrodynamic capacity by yielding a more fusiform shape, thereby decreasing the energetic costs associated with motion or static behaviour in the flow. The effect of the environment was so strong that the slender deformation occurred whatever of the genotype, illustrating the way in which the environment can guide the ontogenetic trajectory of the species body shape in a river, particularly in terms of an upward movement of the pelvic fin landmark.

Another major characteristic of the Durance River is substratum clogging, which is routinely identified as a potential cause of benthic community disturbance [63] particularly for benthic fishes [64]. In addition, it has been shown that perilithon abundance is negatively correlated with substrate clogging in the Durance particularly in the middle part of the river [65] which is characterized by highly regulated flows (see Fig 5). The annual mean for the invertebrate density ranged from 1,366 to 4,467 ind/m^2^ in the Durance River [54] and from 2,800 to 13,000 ind/m^2^ in the Ardèche River [52]. About chondrostoms in particular, previous studies based on stable isotope analysis have shown more opportunistic feeding behaviour in Durance than in the more trophically specialized reference population [32] or Ardèche populations (S7 Fig). These results suggest that trophic resources may be a limiting factor for the diet of chondrostoms in the Durance, highlighting the importance of substrate clogging. Both species in the Durance River experienced similar ecomorphological displacements, although with different intensities. Based on body shape development in the reference populations, the Durance slender deformation is expected to further affect the *C*. *nasus*. Indeed, in the reference populations, the *C*. *nasus* developed a morphological specialization correlated with ecological specialization, particularly with a more benthic lifestyle. During ontogeny, the downward movement of the mouth, due to changes towards deeper body, is correlated with a straightening of the lower lip [47]. These morphological modifications improved scraping capacity and enhanced the animal’s ability to graze on the perilithon [30,66]. In Durance basin, the environment pressure (changes towards slender body shape) and ontogenetical trajectories (changes towards deeper body shape) act in opposed ways in the case of *C*. *nasus*. Conversely, no such ecomorphological specialization towards a benthic lifestyle occurred in *P*. *toxostoma*. River engineering would therefore be expected to have a greater impact on *C*. *nasus* than on *P*. *toxostoma*.

As highlighted by several authors [67–68], the ontogeny constitutes the cornerstone of adaptation between and within fish species. We demonstrate here that this process is particularly important for the chondrostoms, illustrated by a general convergence towards the same ecomorphological state—the maintenance of juvenile traits (notably shown by morphology)—in the altered environment (Durance basin). Interestingly, a similar pedomorphosis phenomenon has recently been identified as a potential response of freshwater fish species to global warming [69], highlighting the effect of temperature as a strong selective and phenotype-molding factor in fishes.

In order to assess the sustainability of this situation, we analyzed the abundance of the ten most present species (including both chondrostoms) in our seven syntopic stations across time (S2 Text, S8 Fig) based on the electrofishing database (48 freshwater fish taxa and 590 sites) of the French National Agency for Water and Aquatic Environments (ONEMA, [70]). It appears that abundances of *C*. *nasus* and *P*. *toxostoma* are quite stable in the Durance, underlining the persistence of a minimum viable population density in this river. This strongly suggests that the observed traits displacement (i.e. pedomorphosis) for chondrostoms in the Durance River do not constitute the premise of a demographic decline, but rather a phenotype displacement (accommodation and/or adaptation) allowing them to persist in this ecosystem.

### Observed versus expected pattern: insight into the future

We had expected *C*. *nasus* to outperform *P*. *toxostoma* because of its invasive capacities and we had hypothesized that the level of introgression would be higher in the Durance zone than in the Ardeche zone because of the higher level of ecological pressure. Indeed, as noted by Mallet (2005), the frequent examples of hybridisation in nature are often attributed to environmental degradation. However, the results obtained in this study contradicted these hypotheses.

In terms of ecological interactions we found that *C*. *nasus* and *P*. *toxostoma* display similar feeding behaviours, with a majority (53%) of the specimens being omnivorous, which suggests a high inter-species overlapping of prey. However, we did not found any sign of underperformance for the endemic *P*. *toxostoma* (considering coefficient of condition and growth). This tends to show that the invasive species does not represent a threatening ecological competitor to the endemic species in the populations studied. The omnivorous behaviour of *C*. *nasus* is most likely explained by the indirect swallowing of invertebrates during the scraping of the epilithon for diatoms. This contrasts with the biting capture behaviour preferred by *P*. *toxostoma*, which displays active efforts to catch invertebrate preys. Based on a comparison of the invertebrate/diatom biomass balance between species, it was suggested that the difference between the two species diets were of a quantitative rather than qualitative order [30]. Therefore, it remains unknown whether the similar qualitative dietary intakes of the two species correspond to different ratios of prey or not. Indeed, with our method, it was possible to clearly identify omnivorous feeding behaviour but not to discriminate between specimens presenting different ratios of various types of food (e.g. 90% diatoms and 10% invertebrates versus 10% diatoms and 90% invertebrates). Next-generation sequencing (NGS) technology is a promising new semi-quantitative method [71] that would facilitate the discrimination between biting (young specimens of both species and adults *P*. *toxostoma*) and scraping (adults *C*. *nasus*) behaviours.

In terms of genomic interactions, the systematic hybridisation clearly indicated that the genomic porosity between the two species is an intrinsic property of the *Chondrostoma*/*Parachondrostoma* species complex, allowing genomic introgression to occur in both directions, but with a greater intensity of gene flow towards *P*. *toxostoma* (see also [34]) irrespective of the environmental conditions. However, contrary to our expectations, we observed a higher rate of introgression in the less anthropized environment (Ardèche basin). Those results echoed with [72] which suggested that the (supposed positive) relation between environmental pressure and hybridisation rate should be treated with caution. This is particularly true for the *Chondrostoma*/*Parachondrostoma* species complex, which has different hybrid zone profiles in the Rhône basin. However, even if environmental disturbance is not the cause of hybridisation in this complex, it may play a role in determining hybrid zone patterns. Further investigations are necessary to understand to what extent the lower level of hybridisation in the Durance could be a consequence of low chondrostoms density (S8 Fig) or dissociation between reproduction periods [25].

### Implication for conservative purpose

The largest populations of *P*. *toxostoma* in the Rhône basin are currently located in the tributaries. *C*. *nasus*, as the invasive species, was expected to force a displacement of *P*. *toxostoma* from the lower Rhône to areas further upstream, such as the Ardèche and Durance tributaries [30]. However, as illustrated by this study, this distribution could also be impacted directly by the engineering of the Rhône, as it has been shown to be the case for invertebrates [73]. Hybrid specimens migrating in the Rhône River were not detected in our study even though *P*. *toxostoma* was most likely present in the Rhône (for at least a) century, before the river development projects and the creation of navigation channels [74]. Syntopic areas are located in the Rhône tributaries and show hybridisation between *C*. *nasus* and *P*. *toxostoma*, as demonstrated in this study for the Ardèche and Durance basins. We demonstrated however that it is not possible to generalize the interactions between the two species as environment features mainly impact the level and pattern of introgression. These results have crucial implications, particularly concerning future management strategies for endemic species.

The presence of abundant introgressed specimens in the Ardèche River constitutes a potential risk to the genomic integrity of the native species. However, the present work demonstrates that the majority of the specimens of this Rhône tributary were « pure » and healthy *P*. *toxostoma*. Thus, even after one century of contact, assortative mating seems to ensure the maintenance of non-introgressed individuals of the native species in the Ardèche. Hence, there seems to be little risk of complete genomic dilution by *C*. *nasus* alleles and even less of a risk of extinction of *P*. *toxostoma* by introgression [7, 75] in the Ardèche basin. Nevertheless, temporal monitoring programs are required at these stations to ensure the stability of hybridisation dynamics and the sustainability of a pure *P*. *toxostoma* population.

Results obtained on the Durance basin demonstrated the huge impact of habitat disturbing on the two species and their hybrids. In the fragmented context, the hybridisation level is not higher than in less impacted ecosystem (Ardèche basin) but environmental pressures are strong enough to constraint all the categories of the complex (*i*.*e*. the two species and their hybrids) towards convergent modifications of phenotypes and diet behaviours. The Durance River served as a refuge for the rheophilic cyprinids of the Rhône basin during the last period of glaciations [76], emphasizing the importance of the preservation of the endemic species' genetic polymorphism for long-term conservation efforts. It was advocated that a peak flow regime allows reconfiguring channel and floodplain habitats in regulated rivers [77]. Attempts to rehabilitate the Durance over the last 10 years have focused on increasing the regulated in-stream flow. This strategy should enhance habitat diversity, which will improve the environmental conditions for the whole fish community, including the non-native species *C*. *nasus*. However, several studies have shown that the natural flow regime (amplitude, volume and duration) may favor endemic species [78–82]. This strategy should be particularly relevant in the Mediterranean context, where species had to adapt to extreme flow amplitudes [83]. Hence, the natural flow regime appears to be the most valuable compromise for river restoration politics in the Durance basin by potentially favoring the endemic species, or at least not hindering them.

## Conclusion

Firstly, *C*. *nasus* was expected to outperform *P*. *toxostoma* because of its invasive capacities. Our study clearly rejects this hypothesis: the two species use distinct spatial habitats in the non-disturbed area and *C*. *nasus* is more affected by environmental pressure in the disturbed area. Secondly, the level of introgression and the hybridisation intensity was not positively correlated with disturbance level as it was expected. Rather, we suggest that the population density and dissociation between reproduction periods could be the main factor that explains the hybridisation process. Thirdly, and contrary to our assumption again, a majority of *P*. *toxostoma* specimens possesses a non introgressed genome, suggesting that assortative mating is sufficiently strong to limit the risk of extinction by hybridisation. Finally, we observed an unexepected ecomorphological traits displacement towards a more juvenile state in all chondrostoms inhabiting in the disturbed area, probably due to environmental pressure.

Hence, this work contributes to shedding a new light on the status of *C*. *nasus*. Indeed, due to its ecological features and particularly its specialist trophic behaviour focused on primary producer consumption, *C*. *nasus* inhabits an ecological niche rarely occupied by other European fishes. On this basis, this species has been considered an ecological opportunity rather than a problem [30], preventing eutrophication by limiting the development of green algae. Our results support this conclusion, as we obtained no clear evidence that *C*. *nasus* constitutes a nuisance for the endemic species *P*. *toxostoma*, consistent with the findings of [30] and other theoretical studies [84]. This is a striking reminder that conservationists and scientists should not “…judge species on their origin” but on their actual and carefully assessed environmental impact [85]. However, further studies focusing on temporal changes in a broad range of syntopic zones should be carried out to demonstrate with certainty the integration of *C*. *nasus* in the ecosystem.

## Supporting Information

S1 Fig Comparison between h-index and q-score. For all of the seven Ardèche and Durance stations (1,550 specimens): q-score (from Structure 2.3 [47]) analysis)) as a function of the h-index (from INTROGRESS analysis) with 0 corresponding to *C*. *nasus* and 1 to *P*. *toxostoma*. Pearson's product-moment correlation r = 0.999 (t = 1252.425, df = 1548, p-value < 2.2e-16). For Structure analysis, the ‘admixture model’ and the “I-model” (independent allele frequencies) were considered, with a burn-in length equal to 100,000 followed by 1,000,000 iterations within a Markov Chain Monte Carlo (MCMC). Polymorphism shared by the two species was taken into account by performing the Introgress analysis, first on allopatric populations and then on allopatric + allotopic populations, as described by [38]. This made it possible to define ranges of *h* corresponding to pure specimens: *C*. *nasus* specimens had *h* values in the range [0, 0.086], whereas *P*. *toxostoma* had *h* values in the range [0.934, 1]. We therefore assigned individuals to three classes, as follows: *Cn* if *h* was in the range [0, 0.086], *Pt* if *h* was in the range [0.934, 1] and Hybrid if *h* was in the range (0.086, 0.934). A nuclear Cn (resp. Pt) individual presenting Pt (resp. Cn) mtDNA will be considered as hybrid.
(EPS)

S2 Fig Hybrid index (*h*) box-plot for the hybrid specimens from each of the seven syntopic populations. A-G. *Y*-axis extending from *h* = 0 (*C*. *nasus*) to h = 1 (*P*. *toxostoma*), n = number of hybrid specimens, the dotted line represents the *h* = 0.5 value. H, Stacked bar chart showing the percentages of the three genomic groups (green = *C*. *nasus*; red = *P*. *toxostoma*; Blue = hybrids).
(EPS)

S3 Fig Coefficient of condition *K*. A) Observed *K* values (in grey) and modeled *K* trajectories for pure specimens (see text for definition) as a function of size (in cm), for reference populations (allopatric+allotropic) and syntopic zones: green disk for *C*. *nasus* and red disk for *P*. *toxostoma*. Black circles correspond to reference populations, blue circles to the Ardèche syntopic zone and orange circles to the Durance syntopic zone. B) Predicted (in black) *K* values and values adjusted (grey) as a function of *h* index from pure *C*. *nasus* (*h* = 0) to pure *P*. *toxostoma* (*h* = 1). Square outlined in blue for the Ardèche basin and in orange for the Durance basin. Square size is proportional to specimen size.
(EPS)

S4 Fig Feces presence in relation to hybrid index. Probability of a specimen containing feces (*y*-axis) as a function of hybrid index (*x*-axis), from a pure *C*. *nasus* (h = 0) to a pure *P*. *toxostoma* (*h* = 1). Reference populations (allopatric+allotropic) are shown in black, Ardèche basin populations are shown in blue and Durance basin populations are shown in orange. The sampling period is indicated by symbols, with circles corresponding to spring, triangles to summer and crosses to autumn.
(EPS)

S5 Fig Body shape for non-hybrid specimens. A) Position of the 21 body landmarks on a specimen. For figures B) to G), significant deformations are indicated by colored arrows; non-significant deformations are indicated in grey and specimens are compared as a function of size, species and environment. B) Mean ontogenetic deformation from the first decile (q1 = 10 cm) to the last decile (q9 = 21.5 cm) C) Mean species deformation for the median size (14 cm) D) Mean ontogenetic deformation by species (green *C*. *nasus*, red *P*. *toxostoma*) from the first decile (q1 = 10 cm for both *C*. *nasus* and *P*. *toxostoma*) to the last decile (q9 = 32.4 cm for *C*. *nasus* and 18.5 cm for *P*. *toxostoma*). E) Mean environment deformation for the median size (14 cm), from reference populations (allopatric+allotropic) to Durance basin (orange) and Ardèche basin (blue) populations. If only one color is shown (e.g. orange for the Durance basin), this indicates that the deformation from reference populations is not significant for the other basin (e.g. blue for the Ardèche basin). When only a grey arrow is shown, none of the comparisons was significant. F) Mean ontogenetic deformation for each of the three environments (reference populations, the Ardèche basin and the Durance basin) from the first decile (reference populations = 12.5 cm, Durance basin = 9.25 cm, Ardèche basin = 10.5 cm) to the last decile (reference populations = 27.38 cm, Durance basin = 19 cm, Ardèche basin = 21.25 cm). Reference populations are shown in black, Durance basin populations in orange and Ardèche basin populations in blue. Grey indicates non-significant ontogenetic trajectories between the three environments. If one color is omitted (e.g. blue for the Ardeche basin), this indicates that the comparison with reference populations is non-significant. G) Mean deformation between species for each environment (reference populations are shown in black, Durance basin populations are shown in orange and Ardèche basin populations are shown in blue). When populations from a syntopic environment (Ardèche basin or Durance basin) do not differ significantly from reference populations, they are shown in black.
(EPS)

S6 Fig Morphological comparisons of the body shapes of hybrids. Shape changes are characterized by displacement vectors for 21 body landmarks and specimens are compared as a function of their syntopic origin (Ardèche basin and Durance basin) and with the morphology expected on the basis of their genomic dilution. A) illustrates the deformation from an expected hybrid (predicted from a pure specimen inhabiting the same syntopic zone) with an *h* = 0.54, with the observed corresponding hybrids, for the Ardèche basin in blue and the Durance basin in orange. An *h*-index of 0 corresponds to pure *C*. *nasus* and an *h*-index of 1 corresponds to pure *P*. *toxostoma*. B) illustrates the expected deformation (blue arrows) and the observed deformation (black arrows) of hybrids of 27.8 cm as a function of their genomic dilution in the Ardèche basin whereas C) corresponds to a similar analysis for Durance hybrids, with the expected deformation vectors in orange. In B) and C), the deformation corresponds to a comparison of hybrids with an *h*-index of 0.47 with hybrids with an *h*-index of 0.90.
(EPS)

S7 Fig Stable isotope analysis. δ13C- δ15N biplots. Stable isotope analysis. δ13C- δ15N biplots with individuals plotted on the basis of their stable isotope signatures. This figure is based on published data [36], to which new points have been added, corresponding to specimens from the three stations from the Ardèche basin, *C*. *nasus* specimens from allopatric populations (Chée and Allier) and *P*. *toxostoma* specimens from allopatric populations (Orbieu). Closed circles in color: red for *P*. *toxostoma*, green for *C*. *nasus*, black for hybrids; open circle color: orange for specimens from syntopic zones (Ardèche basin and Durance basin). Orange circumferences correspond to the Durance basin; blue circumferences correspond to the three stations of the Ardèche basin.
(EPS)

S8 Fig Fitted densities of the 10 most present species. For each of the 10 most present species (number of specimens by 100 m^2^) considering our sampling stations (or proxi): densities values and fitted values by the selected model (see S2 Text). In orange: Durance, in blue Ardèche, in black Rhodanian stations outside Durance and Ardèche. Species names: “CHE” (*Squalius cephalus*), “HOT” (*Chondrostoma nasus*), “SPI” (*Alburnoides bipunctatus*), “VAI” (*Phoxinus phoxinus*), “GOU” (*Gobio gobio*), “ABL” (*Alburnus alburnus*), “LOF” (*Barbatula barbatula*), “BAF” (*Barbus barbus*), “BLN” (*Telestes souffia*), and “TOX” (*Parachondrostoma toxostoma*).
(EPS)

S1 Table Characterization of the eight prey items considered during diet analysis. This table illustrates how the prey items were established from the prey targeted by the 21 group-specific primer sets during the molecular analysis of the feces.
(XLSX)

S2 Table Complete dataset available for this study. (XLS)

S3 Table Table of coefficients for the body size model. A model was established for the assessment of the difference between syntopic and reference populations. In (a), the total syntopic dataset was considered; in (b), specimens from Saint-Just (Ardèche basin) and Avignon (Durance basin) were removed because of the size-bias population at these stations.
(XLSX)

S4 Table Table of coefficients for the coefficient of condition (*K*) model (see text), including the effects of species, size and environment and their interactions. This model was established with a dataset restricted to pure specimens (allopatry and sympatry). In (b), the dataset was restricted to sympatric specimens. Hybrids, characterized by genomic dilution levels, the *h*-index, were analyzed by comparing the values expected assuming a linear mixture of pure specimens living in the same zone (i.e. Ardèche basin or Durance basin) with the observed values.
(XLSX)

S5 Table ANOVA table for the GuVac analysis. (XLSX)

S1 Text Modelling morphological coordinates. (DOCX)

S2 Text Densities modelling of the 10 most present species. (DOCX)
