# Supplementary material for: When Anthropogenic River Disturbance Decreases Hybridisation between Non-Native and Endemic Cyprinids and Drives an Ecomorphological Displacement towards Juvenile State in Both Species
Source: PLoS One. 2015 Nov 11;10(11):e0142592. doi: 10.1371/journal.pone.0142592 (PMC4641742; doi:10.1371/journal.pone.0142592)
Supplement: S1 Text — (DOCX) [file pone.0142592.s014.docx]

Each morphological coordinate was modeled considering two datasets. The first data set (n = 1684) corresponds to individuals defined as non-hybrid (i.e. defined as *Pt* or *Cn*). These individuals were sampled in allopatric and sympatric conditions. The second data set (n = 132) included hybrid specimens in the Ardèche and Durance basins.

*First data set analysis -* We investigated the effects on morphology of three factors, environment (env), size and species (sp), by modelling each coordinate z (constituted by x and y) with the following three-way complete model: z = env x bs(size, m) x sp + ε, where *bs (size, m)* is the polynomial basis of degree equal to *m* = 5 for size. We then considered all the possible submodels ( = 300) built with lower values of *m* (*m = 1* to *5*) and/or grouping environments (i.e. Ardèche-Durance versus “reference populations”, Ardèche-“reference populations” versus Durance and Durance-“reference populations” versus Ardèche) and/or omitting interactions or simple terms from the complete model. For each landmark, we selected the model minimizing the mean AIC (on the two coordinates). Within this model, we consider a type III procedure in order to test the effects of factors. The interpretation of simple effects was simplified by centering the size variable on the overall median (14 cm). For example, species comparisons were carried out for a fixed size of 14 cm.

*Second data set analysis -* Hybrid specimens were genetically defined by *h* and could be seen as a mixture of *P. toxostoma* (proportion *h*) and *C. nasus* (proportion 1-*h*) with similar characteristics (size, environment). We compared the observed morphology with that predicted, assuming that the morphology of the hybrid is a linear mixture of the morphologies of the two pure species. For each coordinate, the difference *D* between the two values was evaluated as follows: D = env x bs(size, m) x bs(h, l) + ε, l, m *= 1,2,3*. The environment variable here can take only two values, Ardèche and Durance, and the second data set is restricted to hybrid specimens. Using the same selection procedure as for first data set analysis, we selected the best model for each coordinate.
