# Supplementary material for: When Anthropogenic River Disturbance Decreases Hybridisation between Non-Native and Endemic Cyprinids and Drives an Ecomorphological Displacement towards Juvenile State in Both Species
Source: PLoS One. 2015 Nov 11;10(11):e0142592. doi: 10.1371/journal.pone.0142592 (PMC4641742; doi:10.1371/journal.pone.0142592)
Supplement: S2 Text — (DOCX) [file pone.0142592.s015.docx]

From Onema's fish database (http://www.image.eaufrance.fr/poisson/cours/p-ce-resultats.htm, last access on 03/04/2015), we selected the sites (n = 15,335) related to the Rhône basin. Sites were classified into three classes: Ardeche basin, Durance basin or other. Considering our sampling station (or proxi), we selected the 10 most present species (number of specimens by 100 m^2^): “CHE” (*Squalius cephalus*), “HOT” (*Chondrostoma nasus*), “SPI” (*Alburnoides bipunctatus*), “VAI” (*Phoxinus phoxinus*), “GOU” (*Gobio gobio*), “ABL” (*Alburnus alburnus*), “LOF” (*Barbatula barbatula*), “BAF” (*Barbus barbus*), “BLN” (*Telestes souffia*), and “TOX” (*Parachondrostoma toxostoma*). For each of these species, we modeled the density (number of specimens by 100 m^2^) as follow: log(dens) = bs(year, knots = 2,deg = k)*zone where *bs (an,deg)* is a piecewise polynomial of degree equal to k with for year with k = 3 to 8. Based on Akaike's criterion, the best model was then selected.
